# Annual Address Delivered to the Graduating Class of the Atlanta Medical College

**Published:** 1888-04

**Authors:** Isaac S. Hopkins

**Affiliations:** President of Emory College, Oxford, Ga.


VOL. V. APRIL, 1888. NO. 2.

### Original Communications.

### ANNUAL ADDRESS DELIVERED TO THE GRADUATING CLASS OF THE ATLANTA MEDICAL COLLEGE.

BY REV. ISAAC S. HOPKINS, M. D., D. D., PH. D., PRESIDENT OF EMORY COLLEGE, OXFORD, GA.

GENTLEMEN—I accept with great pleasure the post of honor assigned me to-night. To deal with young men has been my life-long work, and to speak to them on occasions similar to this is one of my official duties. There are some things, however, which difference this occasion from those with which I am familiar and which invest it with peculiar responsibility and difficulty. By the action of your faculty and the board of trustees of this institution you stand invested to-night with a new and very solemn responsibility. It is true, every epoch in one's life is the introduction to a new responsibility, but this of yours, I repeat, is one of the gravest import. You go hence to represent a widely-known and highly-honored school of medicine. You are legally commissioned to practice a great profession. You are formally charged with the care of that which men universally cherish as of most worth, human life. Looking at the labors you have discharged in the attainment of your present position and the honor you have won, it is in my heart to offer you my sincere congratulation. Looking at your new relationship, the duties it involves, the trials it will bring, the dangers incident to it, it is fitting that I embrace the opportunity to offer you some friendly words of counsel and encouragement.

It would seem idle and superfluous to speak of the honorable character of the profession upon which you have entered. Measured by any standard, that of the names associated with its histories, that of utility, that of emolument, that of dignity and public esteem, it has by common consent ever held a place as one of the three professions most honored by men.

And yet it seems to your speaker that the profession of medicine has not received that measure of honor, or I might better say that peculiar honor to which it is entitled, and which would have come to it from a clearer apprehension of its true scope and aims. It stands differenced from all other professions in a peculiar way—that is in the universality of its relationship to men and things. And this relationship is not casual or incidental—it is positive and official. The profession of medicine embraces things natural and supernatural, things material and immaterial, things social and individual. Like the fabled Briareus, it is the child of heaven and the earth, and holds in its hundred hands untold interests and inestimable treasures. It stands on the border-land between the realm of the senses and that of invisible things, and claims equal dominion in both—it is at once an art, a science and a philosophy.

As an art it had its birth in the necessities of man and finds its limitations in the order of nature. These necessities are pressing now, as they have ever pressed, upon men in the forms of pain and disease. The limitations it shares in common with all art and subordination to nature is its first and fundamental law. Nature furnishes material and agencies in unstinted abundance, but demands in return strict compliance with her own order of things.

And her order with reference to man is an order of health, not an order of disease. Diseased bodily conditions grow out of and are but another name for departures from natural bodily conditions. These departures may have been made centuries before their victim is born, but as soon as discovered nature sets herself to amend and correct. Modern medicine gives no color to the superstition which would hide a disease in every cleft and crevice of the earth, lying in ambush waiting for a victim. Such a conception may serve a good purpose in poetry, or for the eloquent periods of the orator, but it does not answer to hard facts.

Modern medicine gives no encouragement to that class of weeping philosophers, medical and otherwise, who mourn over a long list of unavoidable diseases—"the ills which flesh is heir to." It does not tolerate that conception of diseases which leaves them outside the pale of law, as if they were the caprices of some demon, or the results of blind chance. In the better conception of one of your profession, "The strength of modern therapeutics lies in the clearer perception than formerly of the great truth that diseases are but perverted life processes, and have their natural history, not only a beginning, but a period of culmination and decline. In common inflammatory affections this is now admitted to be almost universal law. By time and rest that innate *vis medicatrix*,

> "Which hath an operation more divine
> Than breath or pen can give expression to,"

reduces the perversions back again to the physiological limits and health is restored. To this beneficent law we owe the maintenance of the form and beauty of our race in the presence of so much that tends to spoil and degrade it. We cannot pass through the crowded streets and alleys of our cities without recognizing proofs of this in the children's faces in spite of all their squalor and misery; and when we remember what this illustration, in all its details, reveals, we may well take heart even when our work seems most hopeless. "*The effects of disease may be for a third or a fourth generation, but the laws of health are for a thousand.*"

In the pursuit of his profession as an art, the physician should rest with supreme satisfaction in the fact that he is called to act in harmony with laws which, if they are inexorable, are, at the same time, beneficent, that the well-nigh omnipotent forces of nature are behind him, and her agencies are his to command.

Inventors have found their highest inspiration in her models. Smeaton found the type for Eddystone Lighthouse in the sturdy build and branching anchorage of the oak tree. Dolland fashioned his achromatic lenses on the model of a human eye. Duhamel braced his bridges in accordance with a suggestion derived from a human shin-bone.

It has been said of the Romans that their art of building bridges and aqueducts was a nature working to municipal ends. So it must be of medical art—a nature working to remedial ends.

But I have said that medicine is a science as well as an art. The two stand very closely related to each other, but for our purpose need to be distinguished. Art, in the earlier stages, is anterior to science. It may afterwards borrow art from it. The object of science is knowledge; the object of art is method· Science is a body of principles and deductions to explain the nature of some object-matter. An art is a body of precepts, not practical skill for the completion of some work. A science teaches us to know, an art to do. Science gives principles, art gives rules. Science evolves the principle which art involves.

Medicine has been defined to be the science of disease and its remedies. The definition may do for a working hypothesis, but as an expression of the full truth concerning the scope and aim of medicine, it is bare and narrow. It were better to call it the science of health and its promoters. But however we may limit it by definitions, this science, this knowledge refuses to be limited in fact, and gathers in its broad sweep the elements of many sciences, and lays them under tribute to enrich its own field and increase its own powers. In the body of man it deals with the same forces which find their mightier but not more beautiful display in the storm-cloud and the sunshine. In this compact laboratory it takes cognizance of the same agencies, of the same beautiful interchange of atoms, which the chemist repeats and imitates in his crude and clumsy contrivances of retort and reagent. It finds apparatus, mechanical, physical, chemical, ready to its hand for study and experiment—such apparatus as a magician might court and which no recreant handling can impair. Turning from these it finds forces of infinite subtlety playing between the gross matter, which is but a machine, and the lowly spirit which is its master. It scans with unwearying patience the glistening net-work of communication scattered throughout this microcosm, and is never discouraged because it cannot surprise the swift and silent courier out of the secret of his power. It follows the ever-flowing stream of life through channels and windings more bewildering than any labyrinth of Moeris or Crete seeking to pene-trate the mystery of life which mingles with its tides. Nor is this science of medicine confined to the individual man. It throws men into classes according to temperament, latitude and nation-ality, and pushes its investigations along lines which are fixed by suffering and poverty and crime. It claims an interest in the domestic and social departments of human life, and fixes principles which themselves underlie the foundations of families, nationali-ties and governments. A strange protean science is this, equally at home in the hovels of the poor, the mansions of the rich, the hospitals of the sick, the prison-house of the despairing, the asylum of the insane, the convocations of cabinets, the audience-chambers of kings. In each it gathers new stores of knowledge and to each it offers the fruit of its labor that men may learn by happy experience the blessing of universal health.

But I have said that medicine is a philosophy, and so it is in that modified but very important sense in which we commonly speak of a philosophy. It has much to say in that department of thought by which the relation of man's body to his soul is defined. It traces the marks of moral evil upon the body as theology traces these marks upon the soul. It essays the problem of natural heritage of traits physical and moral. It traces the path of pestilence from continent to continent and measures the physical evils which fol-low in the track of war. It claims to have seen in mental affec-tions the tendency to contagion, and has sought to settle the con-ditions and methods of cure for such epidemics. It offers its counsel to learned courts on the guilt or innocence of the prisoner at the bar, assuming to decide such questions on grounds physiological and psychological. It has, in some of its votaries at least, been bold enough to propose its own theory of mental action, and now even claims to measure the degree of intellectual vigor by the waste products evolved.

If these principles be just and true, several deductions grow naturally and directly out of them—deductions which may in some measure be a guide to you in shaping your studies and directing your energies as you follow the inviting paths opening before you.

And, first, the outlook of medicine is not in the direction of a multiplication of remedial agents. Already the drug market is overstocked and every day brings before the physician some new candidate for his favor. There is a fashion in drugs almost as alluring as that in hats for the fairer sex. Hardly a decade passes that does not witness the rise, culmination and decline of some popular and specific remedy. Out of the multitude some remain on the principle of the survival of the fittest. This principle of elimination has gone on through the centuries, proving, not the worthlessness of drugs, but full of suggestions in the line indicated, that advance is not in this direction. Many foolish and hurtful things have been said in diversion, and some in dead earnest, about the inefficacy of remedial agents. Perhaps much harm has been done by the unwisdom with which physic has been prescribed, but, in all seriousness, it is suggestive enough that physicians, who grow wiser as they grow older, give less physic and more counsel, push the expectant method and lose fewer patients by death or otherwise.

In a matter so purely professional, it may be well for a layman to be economical of his counsel, but he may at least venture to suggest to young gentlemen, first entering a medical career, the experiment of prescribing, in safe cases of course, a treatment of base ball and bycicle tournament in preference to jaborandi, pilocarpine and chloral hydrate.

A more promising field of medical growth is to be in the study of disease in its origin and development, and this field embraces the broadest and most varied pursuits and investigations. Anatomy has changed base in these later years, and, under the guise of a new and more comprehensive science, invites the student, with scalpel and microscope, reagents and battery, to get ready for the better understanding of the relations of structure and function.

Physiology has not yet yielded all its secrets, and waits for acute observers, writing on every portal and column of her fair temple: "Here a man may see whatever he brings an eye to see."

Physical appliances for investigation of exquisite ingenuity and beautiful simplicity have made diagnosis almost a new science. True, one is a little bewildered who comes out of an antique past into a modern doctor's office and sees his armament of ophthalmoscopes and rhinoscopes and otoscopes and laryngoscopes and stethoscopes and thermometers and spirometers and dynamometers and sphygmometers and pleximeters and probes and probangs. But "the tools to the man who can use them."

New and startling theories of the origin of disease leak out of the medical arcana to which the uninitiated in these mysteries give much attention. Lister tells us that his antiseptic gauze and vapor excludes or destroys floating germs, microbes, bacilli or what-nots which thicken the atmosphere and hover over every open wound, threatening to inoculate and poison the blood. Koch tells us he has really discovered the peculiar form of microscopic monster, which, propagated in the lungs, give rise to that scourge of the human race, tubercular consumption.

If these claims are well founded, how vast a field of research and discovery is here opened for the patient toiler and faithful investigator.

But again, a wide-open door is found in that fact which gave to men of the medical profession the name by which they are best known—the name of doctor. The word means teacher. The question would bear consideration if the highest office of any profession is not in what it teaches rather than in anything else it proposes to do. I am sure it is so in this profession.

Beyond the alleviation of pain, beyond the healing of sickness, there is a value which is found only in the beneficent work of teaching men the glory and beauty of life, of leading them into such knowledge of their being as to avoid the causes of pain, prolonging life, staying the march of disease, teaching men how not to be sick.

Young gentlemen, if this brief and imperfect outline of the claims and objects of your profession is at all true, the pressing question of this hour is, what manner of man ought a doctor to be, and to this question I now invite your attention.

In the first place he ought to be a man of vigorous health. A doctor can hardly afford to be sick. Apart from the disabilities his sickness would entail upon his patients, he ought to illustrate in his own person the blessedness of that skill by which he proposes to bring health to others. The foundation of all success in physical and intellectual labor, I need hardly remind you, is laid in a healthy, vigorous body. The apparent impertinence of the counsel I am sure you will excuse when I say that the doctor who breaks down in health does so either from gross carelessness of the laws of health as he teaches them to others, or else from a mistaken conception of what is due to the claims of men upon his time and energies. It is doubtful if any interpretation of his duty would warrant such a tax upon his strength as to disqualify him for further service. It is certain that prodigal wastefulness of his energies, either by pernicious habits of life or professional labors, is little less than a crime.

But, again, as contrasted rather than opposed to this view, the doctor should be a man of heroic mould. There are times when he cannot spare himself. When those to whom he stands pledged, professionally, are in sore need and extremity, a need and extremity greater than his own, when the dark wing of pestilence broods over the land, when the epidemic is desolating homes and wrecking the happiness of communities, when defenseless womanhood and helpless infancy together demand his instant service at such times, to put his own comfort or his own health and safety in the balance against the demands of the sick and suffering is dastardly and base. The man who has not the nerve to stand unflinchingly before the contagious and pestilential foe when it invades the field of his professional labors has no business in the profession.

Two classes of men of all others are called to brave the perils of contagion and smile at the dire portent of the pestilence—the preacher and the doctor—and to the credit of humanity, be it said, that in both classes the record has furnished rare exceptions to the rule of heroism and duty.

These thoughts naturally enough suggest another requisite of the doctor's character. He should be a man of large, broad, intelligent sympathy. One would think that in the presence of so much suffering he could hardly be otherwise, but the very occasion of his sympathies is the source of his peril. I do not mean that he should be a maudlin sentimentalist, mourning over the ills of his patients when he ought to be thinking how to cure them, hesitating in the choice of his treatment because a dose is nauseous, or an operation painful, but that he should be a man with a heart in him responsive to the claims for pity and help, with a skill at the service of the humble and lowly as well as the rich and great, with an interest in the whims even of his hysterical patient and entering into the thought and feelings of little children. It is one of the unexplained things in mental operations how sympathy promotes intelligence and how quiet, responsive feeling aids intellectual activity.

And no man needs more than the doctor all his intellectual powers in the highest degee of activity. For the ordinary duties of his calling he needs the cool, clear, incisive intellect—the mind not only stored with the treasures of knowledge, but able to follow in consecutive, logical order the leadings of truth, linking fact to fact and inference to inference with unhesitating and unerring certainty. There are times when the scale of destiny and the issues of life hang upon the quickness of his perceptions and the truthfulness of his intuitions. One of the most significant and marked differences between men is found in this ability to think rapidly and vigorously under the pressure of an emergency. Such ability is not born in a moment—it is the outcome of the fullest training of the mind under ordinary calls for thinking. Like the trained steed or athlete, with nerve and muscle in tone, active, alert, prompt when the occasion arises for the extraordinary leap or run, the trained muscle yields a ready response and the unusual feat seems but a holiday pastime.

I know no better counsel that I could give you in this connection than to urge you to spare no effort to keep your perceptive and intuitive powers up to their highest point of efficiency. We have all known physicians in whose presence we were almost afraid to think, so keenly alive were they to the things transpiring around them, so tense and active were their sense perceptions, so well trained their powers of observation. Such qualities as these can come only from a tireless industry and unquestioning obedience to the laws of your profession.

But there are moral qualities, not embraced in anything I have said, which are essential to the physician.

They may be summed up in the statement that only the man of pure mind and untarnished character is fit to be a doctor. The sacred confidences of the sick-room, his freedom of access to the sanctuary of the home, the intimate personal relations to all ages and both sexes, and every condition of men, call for an exalted moral excellence in the physician which is not rivalled by any other calling, except that of the gospel minister, and is not transcended by his. By every token of usefulness and honor, of character and purpose, the profession you have chosen demands a life free from vice, a name above reproach, "a bosom that harbors no dangerous secrets and a heart that might be turned inside out and discover no stain of dishonor."

But finally, young gentlemen, the drift of all this counsel may be summed up in one word: The doctor must be a man obedient to the law he imposes upon his patient, and in such obedience he will find that to which his professional character even must be subordinate, a true and worthy manhood, a likeness to Him who went about doing good, and whom men have loved to honor through the ages as the Great Physician.

