# Errata

**Published:** 1888-04

**Authors:** 


ERRATA.

Page 69, second line, for "court" read "covet;" for "recreant" read "reverent." Fourth lin e, for "lowly" read "lofty."

